# Supplementary material for: Content-rich biological network constructed by mining PubMed abstracts
Source: BMC Bioinformatics. 2004 Oct 8;5:147. doi: 10.1186/1471-2105-5-147 (PMC528731; doi:10.1186/1471-2105-5-147)
Supplement: Additional File 5 — The original Chilibot query results of the term "long-term potentiation (LTP)" and 22 other terms, limiting the latest references analyzed to the years 1990, 1995, 2000, and 2004. [file 1471-2105-5-147-S5.bz2 › chilibotAdditionalFile5/ltp1995/html/NMDA_ZIF268.html]

 


 **NMDA** and **ZIF268** 
  
Found 2 abstracts in PubMed,  **2 abstracts were retrieved and analyzed**.  


---

 Search Google  |
 PDF files only 
|  EDU domain only 

---

- J Neurosci, 1993   **Thresholds for synaptic activation of transcription factors in hippocampus correlation with long term enhancement.** .
  Recent studies suggest a role for rapid induction of transcription factors in stimulus induced neuronal plasticity in the mammalian brain.
  Synaptic activation of transcription factors has been analyzed in the hippocampus using the long term potentiation or enhancement LTP LTE paradigm.
  Using this approach, several studies have identified transcription factors that are induced in hippocampal granule cells by **NMDA** receptor dependent mechanisms.
  the link between long term plasticity and activation of these genes has been called into question by reports suggesting that the thresholds for LTE and gene activation differ.
  To address this issue, we have used a chronic in vivo recording technique to monitor mRNA responses of several transcription factor genes to two different patterns of LTE inducing electrical stimulation of entorhinal cortical afferents to hippocampus.
  One pattern consisted of 10 repetitions of a 20 or 25 msec train of pulses at 400 Hz 80 or 100 pulses total .
  This 10 train pattern has been used in previous studies of LTE and produces robust synaptic enhancement lasting at least 3 d Barnes, 1979 .
  The other stimulation pattern consisted of 50 repetitions of a 20 msec train delivered at 400 Hz 400 pulses total , which is similar to parameters used in other studies reporting induction of c fos in association with LTE Dragunow et al.
  , 1989.
  Jeffery et al.
  , 1990.
  Abraham et al.
  , 1992 .
  Our results indicate that expression of **zif268**, monitored by in situ hybridization and immunostaining, is strongly induced by the 10 train stimulus pattern to levels similar to those induced by seizure activity.
  JunB mRNA levels are also modestly increased by the 10 train stimulus pattern.
  increases in JunB immunostaining were not detected.
  Neither c fos nor c jun mRNA were detectably induced by this stimulus.
  In contrast, the 50 train stimulus pattern resulted in a robust induction of c fos and c jun mRNA, in addition to **zif268** and junB.
  Transcription factor responses to either stimulus pattern were blocked by the noncompetitive **NMDA** receptor antagonist MK 801.
  Identical transcription factor responses were observed in adult 6 12 month old and aged 23 26 month old rats, suggesting that synaptic mechanisms involved in these responses are preserved in aged animals.
  Analysis of LTE following either the 10 or 50 train stimulus patterns revealed identical magnitudes of initial induction and decay kinetics approximately 3 d and indicates that the 10 train stimulus pattern is sufficient to produce maximal synaptic enhancement.
  ABSTRACT TRUNCATED AT 400 WORDS

  - Nature, 1993   **Numerous candidate plasticity related genes revealed by differential cDNA cloning.** .
    Plasticity is a property of the nervous system that allows it to modify its response to an altered input.
    This capacity for change suggests that there are molecular mechanisms in neurons that can couple stimuli to long term alterations in phenotype.
    Neuronal excitation elicits rapid transcriptional activation of several immediate early genes, for example c fos, c jun and **zif268**.
    Many immediate early genes encode transcription factors that control expression of downstream genes whose products are believed to bring about long term plastic changes.
    Here we use a highly sensitive differential complementary DNA cloning procedure to identify genes that may participate in long term plasticity.
    We cloned 52 cDNAs of genes induced by the glutamate analogue kainate in the hippocampus dentate gyrus.
    The number of these candidate plasticity related genes CPGs is estimated to be 500 00.
    One of the cloned CPGs 16C8 , encoding a protease inhibitor, is induced by a stimulus producing long term potentiation and during dentate gyrus development.
    a second, cpg1, is dependent on activation of the **NMDA** N methyl D aspartate receptor for induction and encodes a new small, dentate gyrus specific protein.
    Seventeen of the cloned CPGs encode known proteins, including six suggesting that strong neuronal activation leads to de novo synthesis of vesicular and other synaptic components.
